# Therapeutic approaches targeting seizure networks

**DOI:** 10.3389/fnetp.2024.1441983

**Published:** 2024-08-07

**Authors:** Jenna Langbein, Ujwal Boddeti, Matthew Kreinbrink, Ziam Khan, Ihika Rampalli, Muzna Bachani, Alexander Ksendzovsky

**Affiliations:** ^1^ Department of Neurosurgery, University of Maryland School of Medicine, Baltimore, MD, United States; ^2^ Surgical Neurology Branch, National Institute of Neurological Disorders, National Institutes of Health, Bethesda, MD, United States; ^3^ Department of Neurosurgery, Baylor College of Medicine, Houston, TX, United States

**Keywords:** epilepsy, epilepsy networks, epilepsy surgery, neuromodulation, seizures, seizure networks, resection, ablation

## Abstract

Epilepsy is one of the most common neurological disorders, affecting over 65 million people worldwide. Despite medical management with anti-seizure medications (ASMs), many patients fail to achieve seizure freedom, with over one-third of patients having drug-resistant epilepsy (DRE). Even with surgical management through resective surgery and/or neuromodulatory interventions, over 50 % of patients continue to experience refractory seizures within a year of surgery. Over the past 2 decades, studies have increasingly suggested that treatment failure is likely driven by untreated components of a pathological seizure network, a shift in the classical understanding of epilepsy as a focal disorder. However, this shift in thinking has yet to translate to improved treatments and seizure outcomes in patients. Here, we present a narrative review discussing the process of surgical epilepsy management. We explore current surgical interventions and hypothesized mechanisms behind treatment failure, highlighting evidence of pathologic seizure networks. Finally, we conclude by discussing how the network theory may inform surgical management, guiding the identification and targeting of more appropriate surgical regions. Ultimately, we believe that adapting current surgical practices and neuromodulatory interventions towards targeting seizure networks offers new therapeutic strategies that may improve seizure outcomes in patients suffering from DRE.

## 1 Introduction

Epilepsy is one of the most common neurological disorders, affecting over 65 million people worldwide (Ngugi et al., 2010). Although first-line management involves over 30 available options (Gunasekera et al., 2023). However, around one-third of patients will fail to achieve seizure control despite extensive pharmacotherapy (Löscher and Klein, 2021). If further workup is pursued in these patients, they typically undergo surgical evaluation in epilepsy centers to localize seizure foci for eventual surgical targeting (i.e., resection, neuromodulation) (Lerche, 2020). Yet, despite direct targeting of suspected epileptogenic zones, over 50 % of surgical patients continue to experience refractory seizures, suggesting failure to address the underlying pathophysiology of epilepsy (Wiebe et al., 2001; Tonini et al., 2004; Engel et al., 2012).

The past two decades have seen an increase in intracranial studies suggesting the presence of pathological seizure networks, see Table 1 (Spencer, 2002; Engel et al., 2013a; Lehnertz et al., 2023). These studies lend pivotal support to the network theory of epilepsy, first proposed in 2002 by Dr. Spencer. She defined seizure networks as functionally and structurally connected circuitry, whereby activity in any one area can affect the entire network (Spencer, 2002). Specifically, she proposed that seizure activity entrains neural networks as a whole, and that the classic thought of focal areas of ictal onset is misrepresentative of the true underlying mechanism of disease. Rather, strongly connected networks underlying seizure propagation result in observed seizure phenomena and the persistence of epileptic disorders. This switch in dogma was reflected in the ILAE’s (International League Against Epilepsy) recent revision to their classification system, now defining focal epilepsy as seizures arising in networks isolated in a single cerebral hemisphere, and generalized epilepsy as seizures that rapidly engage with bilaterally distributed networks (Berg et al., 2010). This understanding of seizures and the role of intracranial networks is supported by studies which show that measures outside of seizure onset localization are important in achieving seizure control (Spencer, 1996; Engel et al., 2013b; Andrews et al., 2019). However, despite this shift in understanding and evidence for seizure networks, we have yet to see this translate to improved treatment options and outcomes in patients with DRE.

**TABLE 1:** This table summarizes studies which lend evidence to support the failure to address the underlying epileptic network as a potential mechanism for treatment failure.

	Study	Study characteristics	Key findings
Pharmacotherapy	Kreilkamp 2021	n = 27 Retrospective	Drug-resistant patients have greater alterations in brain networks compared to drug-responsive cohorts at the time of diagnosis
Pharmacotherapy	Tan 2021	n = 37 Prospective	Drug-resistant patients demonstrate higher thalamocortical functional connectivity compared to seizure-free patients
Surgical	Andrews 2019	n = 118 Retrospective	Patients with rapid seizure spread outside the resective zone have higher risk of post-surgical recurrence
Surgical	Hall 2023	n = 22 Prospective	Increased abnormal network connections in extra-temporal epilepsy are associated with persistent seizures
Surgical	Sinha 2021	n = 51 Retrospective	Direct correlation between number of abnormal network nodes and seizure recurrence in anterior temporal lobe epilepsy
Surgical	Neal 2020	n = 19 Prospective	More extensive surgical disconnection of temporal lobe epilepsy networks is associated with decreased seizure recurrence, as well as positive effects on neuropsychological functioning
Neurostimulation	Charlesbois 2022	n = 22 Retrospective	Increased structural connectivity between RNS stimulation site and other brain regions correlates with RNS effectiveness
Neurostimulation	Khambhati 2021	n = 51 Retrospective	RNS responders demonstrate significant reorganization of interictal functional connectivity compared to non-responders
Neurostimulation	Kobayashi 2023	n = 12 Retrospective	Functional connectivity can guide optimal placement of RNS electrodes for improved outcomes

Over the past few years, there have been significant technological advancements, shaping how we treat epilepsy. For example, in 2013, the United States Food and Drug Administration (FDA) approved the first closed-loop neuromodulation device, Responsive Neurostimulation (RNS) made by NeuroPace, that detects electrographic changes preceding seizures and applies counterstimulation to halt seizure activity (Boddeti et al., 2022). However, studies find that the therapeutic benefit seen in some patients is not necessarily due to the immediate seizure disruption, but can be more likely attributed to alterations to chronic network changes (Rao and Rolston, 2023). Although new therapeutic solutions are available, if they are applied using an outdated framework (i.e., epilepsy as a focal disorder), and do not adapt to target seizure networks, seizure outcomes may be suboptimal. Here, we present a review discussing the current state of DRE management, focusing on focal epilepsy due to the larger role of surgical management for focal epilepsy compared to generalized epilepsy (Bullinger et al., 2022). We present recent studies that provide strong evidence of seizure networks in people with epilepsy. Finally, we conclude by discussing how the network theory may guide future surgical epilepsy management.

## 2 Drug-resistant epilepsy management

As discussed above, patients with seizure disorders are typically managed with ASMs (Gunasekera et al., 2023). However, ASMs will fail to achieve seizure control in approximately one-third of patients, warranting a comprehensive tripartite workup elucidating focal targets for subsequent surgical intervention (Lerche, 2020; Kanner and Bicchi, 2022).

Phase 1 employs non-invasive monitoring through a variety of modalities to identify potential focal targets that may be concordant with a patient’s seizure semiology. These modalities include video scalp-electroencephalography (EEG), neuropsychological testing, and functional neuroimaging, such as positron emission tomography (PET), ictal single photon emission computed tomography (SPECT), and functional magnetic resonance imaging (fMRI) (Munakomi and Das, 2023). Workup progresses to phase 2 if non-invasive tests are inconclusive or identified intracranial regions of interest are discordant with seizure semiology. Phase 2 includes neurosurgical implantation of subdural electrode grids and/or stereotactic depth electrodes (sEEGs) to record directly from cortical/subcortical areas and localize seizure onset regions (Baumgartner et al., 2019). After electrode implantation, patients remain in the epilepsy monitoring unit (EMU) and are weaned off ASMs, to allow for recording of seizure activity that can be used to localize seizure targets and inform subsequent surgical treatment (Bagić et al., 2024).

### 2.1 Resective/ablative surgery

Surgical intervention is largely dependent on location of seizure onset. To date, clinical evidence strongly supports resective surgery as superior to medical management for focal epilepsy, especially in patients with DRE (Wiebe et al., 2001; Engel et al., 2012). One retrospective study of 284 patients with focal DRE found that 47 % of patients remain seizure-free 5 years after epilepsy surgery and 38 % remain seizure free after 10 years (Mohan et al., 2018). Even among the patients who experience recurrence following resection, the majority find significant seizure reduction (Yoon et al., 2003; Mohan et al., 2018). Magnetic resonance imaging-guided laser interstitial thermal therapy (MR-guided LITT) is a surgical technique which ablates the target region, similar to a resection, but has the advantage of being less invasive than traditional resection. For MR-guided LITT, a 2023 meta-analysis found that 57.9 % of patients, across all DRE etiologies, were seizure-free at a median 19-month follow-up (Chen et al., 2023).

### 2.2 Neuromodulation

However, a subset of patients may be inappropriate candidates for surgical resection. This can be due to a myriad of factors, including seizure foci localizing near eloquent areas, patients presenting with medical comorbidities, or absence of discrete seizure foci, such as may be seen in generalized epilepsy (Rugg et al., 2020; Munakomi and Das, 2023). In these cases, interventions like neuromodulation devices provide effective alternatives to cortical resection (Guery and Rheims, 2021). A recent meta-analysis reported seizure reduction in 64.8 % of deep brain stimulation and 48.3 % of vagus nerve stimulation patients treated for generalized epilepsy at mean follow-ups of 23.1 and 22.3 months, respectively (Haneef and Skrehot, 2023). In the 9-year Responsive Neurostimulation (RNS) long-term treatment trial, 18.4 % of patients with focal epilepsy experienced at least one period of seizure freedom that lasted a year or longer (Nair et al., 2020). Of this subset, 62 % of patients were seizure free at the last follow up.

Despite the multitude of surgical interventions, and the success of these treatments, a significant proportion of patients do not achieve seizure freedom. Additionally, the mechanisms of treatment failure are still unclear, however commonly reported factors portending treatment failure are incongruent electrophysiology data, non-lesional epilepsy, and ill-defined epileptic focus (Zhang et al., 2013; Krucoff et al., 2017; Chen et al., 2023). These predictive factors suggest that treatment tends to fail when the seizure network either spatially extends or is functionally connected beyond the epileptic focus. Discordant electrophysiology data and an ill-defined epileptic focus suggests perhaps more than one epileptic focus, as would be seen in an epileptic network; non-lesional epilepsy suggests either a structural etiology at too small of a resolution or epileptic activity too diffusely distributed that there is no identifiable lesion on standard workup. Given that many patients continue to experience seizures despite aggressive surgical management, it is imperative to understand the underlying causes of treatment failure.

## 3 Mechanisms of treatment failure

All seizure therapies, from ASMs to surgical intervention, struggle to target the underlying epileptic network, acting either too broadly or too focally. Pharmacotherapy reduces seizure activity but fails to address underlying pathologic network alterations. In fact, the recent push towards defining these medications as “anti-seizure” rather than “anti-epileptic” stems from a recognition that these therapies provide symptomatic treatment only and have not been found to alter the disease course (Löscher and Klein, 2021). Further research aimed at understanding the molecular basis of network formation, as well as developing drugs that target these mechanisms directly, is certainly warranted. Prior studies have shown a correlation between pharmacoresistance and a higher number of alterations across widespread brain networks, compared to the drug-responsive cohort (Kreilkamp et al., 2021; Tan et al., 2021; Sinha et al., 2022). This suggests that patients with DRE have altered networks that persist beyond the management of the anti-seizure medication, though the order of causality - whether network changes worsen due to ineffective medication or networks are inherently unresponsive to the medication - is presently unknown. Network reorganization through adaptive neural processes can reinforce the formation of these abnormal networks (Loscher et al., 2020). Prior studies have shown that hippocampal sclerosis in focal temporal lobe epilepsy is associated with ASM resistance, suggesting that these pathological changes may contribute to ASM treatment failure by altering ASM targets in the brain, leading to reduced drug sensitivity (Loscher et al., 2020). Addressing the broader network alterations in drug-resistant patients may elucidate differences in disease pathophysiology or potential treatment targets avenues for improved outcomes.

In contrast to the broad effects of ASMs, resective surgery targets removal of defined epileptogenic foci. However, this focal approach can prove insufficient. One retrospective study of 57 patients with focal DRE found four major causes of treatment failure: erroneous identification of the epileptogenic foci during workup, intraoperative mistakes in identifying or guidance to the resection zone, generation of new epileptogenic areas, and incomplete resection due to functional limitations (González-Martínez et al., 2007). These findings can in part be explained by an epileptic network which is incompletely identified or targeted with resection. In 2019, Andrews et al. conducted a retrospective study of ECoG recordings obtained from patients with focal DRE during neuromonitoring (Andrews et al., 2019). They found that patients with temporal lobe epilepsy who experienced significantly poorer post-operative seizure outcomes had resections that did not include cortical areas of rapid seizure spread, determined by intracranial EEG ( < 10-s) (Andrews et al., 2019). Considering seizure spread times as a proxy for highly connected brain regions, these findings suggest the importance of resecting areas that play a crucial role in the underlying seizure network to effectively intervene in a patient’s disease course (Shah et al., 2019). Insights into extra-temporal epilepsy show similar findings. Temporal plus epilepsy, the concept of surgically-refractory patients with focal temporal lobe epilepsy demonstrating an epileptogenic zone (EZ) or epileptogenic network which extends into neighboring regions, has a strong association with the risk of seizure relapse following resection (Barba et al., 2016). A recent study found that patients with extra temporal lobe epilepsy who had higher proportion of abnormal connections, determined by comparing the generalized fractional anisotropy (gFA) in diffusion MRI of each connection with corresponding connections in controls, were less likely to be seizure-free (Hall et al., 2023). Failure to surgically address abnormal connections appears to increase risk of seizure recurrence, likely due to the presence of epileptogenic networks persisting despite resection of a focal seizure node. Another study investigating the relationship between brain networks and outcomes of anterior temporal lobe resection found a direct correlation with the number of unresected abnormal network nodes and seizure recurrence, with abnormal network nodes identified using the number of abnormal connections determined by gFA to the area designated as the node, and seizure recurrence. This suggests the significance of modulating the broader seizure network to effectively improve seizure outcomes (Sinha et al., 2021). This finding is bolstered by recent research which correlated decreased rates of seizure recurrence with a greater degree of surgical disconnection of the implicated functional temporal lobe epileptic network as determined by an algorithm combining non-invasive EEG and resting state functional MRI data (Neal et al., 2020). This evidence suggests two key insights: 1) there is a strong connection between surgically-refractory temporal and extra-temporal lobe epilepsy and increased abnormal network connectivity and 2) therapeutic strategies which maximize abnormal network disruption may improve outcomes.

## 4 Identifying and targeting seizure networks

In translating a network approach to clinical practice, the first challenge is to accurately identify the target epileptic network. Imaging to detect seizure networks can be broadly categorized as either structural or functional imaging. Structural imaging depicts the physical connections within a neural network, such as through tractography (Guo et al., 2024). Functional imaging reveals the temporal associations in neuronal activation between different regions of the brain that comprise a network; examples of this include EEG, fMRI, MEG, and PET (Guo et al., 2024). Each modality has advantages and disadvantages, and concerted use can compensate for limitations (Guo et al., 2024). One case report found sustained seizure control after a second resective procedure for non-lesional frontal lobe epilepsy. Analysis of network hubs - defined as regions that play crucial roles in network communication, identified by several different imaging modalities including sEEG, fMRI, and diffusion tensor imaging (DTI) - demonstrated that the second surgery’s resective zone included highly connected hubs that were spared by the first resection (Gong et al., 2024). This underscores the importance of adopting a multimodal approach to surgical planning to adequately identify significant nodes within a seizure network, thereby optimizing seizure reduction outcomes.

Current presurgical planning largely relies on capturing seizure events during invasive and/or noninvasive EEG recordings (Baumgartner et al., 2019). This approach requires significant recording time and may fail to identify suitable targets (Pondal-Sordo et al., 2007; Shah and Mittal, 2014). The recent evidence suggesting seizure activity on EEG can arise from any number of critical nodes in the seizure network may explain neuromonitoring findings that are, at times, discordant with a patient’s seizure semiology (McGonigal, 2020). This implies that a recording-based approach would require capture of many seizure events to characterize the full seizure network (Liu et al., 2022). On the other hand, network-based localization may compensate for these limitations. For example, studies have shown that interictal functional connectivity (FC), determined by EEG, can be used to characterize the seizure network (Lagarde et al., 2022; Shamas et al., 2022; Rijal et al., 2023). As such, it may be possible to identify seizure networks from baseline interictal recordings, obviating the need to capture multiple seizure events. Perhaps a network-based approach to presurgical planning may extend to include measurements of interictal functional connectivity, obtained during the phase 2 SEEG mapping, in order to better characterize the seizure network and aid in precise surgical targeting.

Building on these insights, recent studies have supported the use of additional imaging techniques to elucidate seizure networks to understand post-ablative or post-resective results. For instance, MRgLITT outcomes have been shown to depend on functional connectivity, as measured on resting-state fMRI, to the ablative region. Ablations leading to seizure freedom exhibit more localized functional connectivity compared to ablations with poorer outcomes in patients with focal epilepsy (Mithani et al., 2019). This study also found that the intrinsic connectivity, from resting-state fMRI, of the ablative region was more predictive of post-surgical outcome than the specific anatomical location of the ablative surgery (Mithani et al., 2019). This suggests the potential utility of network characterization for presurgical planning of ablative procedures to ensure sufficient disruption of the seizure network and consequently to improve outcomes.

Additionally, taking a network approach to imaging before neuromodulation may improve therapeutic efficacy (Piper et al., 2022). Currently, RNS targets the epileptic focus identified from pre-operative mapping studies. Studies have recently demonstrated that assessing preoperative seizure networks can prognosticate neurostimulation response, suggesting the possibility of using individual circuit dynamics to help tailor treatments more effectively (Fan et al., 2022; Scheid et al., 2022). One study looked at direct involvement of the network to assess the correlation with treatment response. They found that increased structural connectivity, measured from patient-specific DTI tractography based on diffusion MRI, between the stimulation site and other brain regions correlated with the effectiveness of RNS treatment for patients with focal epilepsy (Charlebois et al., 2022). This suggests that the success of neuromodulation may depend on how stimulation connects to and thereby influences brain networks linked to the stimulation site. Moreover, a recent study found that RNS responders demonstrated significant reorganization of interictal functional connectivity, as determined by intracranial EEG, within neural networks compared to non-responders, suggesting that better treatment response is associated with more effective changes to the network (Khambhati et al., 2021). Additionally, a new study looked at cortico-cortical evoked potentials from intracranial EEG in patients with focal epilepsy and RNS implants and showed that RNS outcomes correlated with placement of electrodes near receiver or projection nodes within the seizure network. This suggests that analysis of functional connectivity may help guide optimal placement of RNS electrodes to improve outcomes, rather than relying solely on ictal electrophysiological changes as is the current standard (Kobayashi et al., 2023).

In the context of deep brain stimulation (DBS), targeting the thalamus is thought to disrupt ictal propagation through prevention of seizure spread to upstream cortical sites, lending support for the notion that the broader network is an important target for epilepsy treatment (Li and Yang, 2017). Emphasizing DBS targets which most greatly impact the network - either due to the target region being highly connected to the rest of the network or the site where multiple circuits converge - may provide an avenue for potential new neuromodulatory targets (Li and Yang, 2017). It is worth noting that while thalamic DBS is intended as a network-based therapy, long-term clinical trials comparing thalamic DBS to RNS, with focal targets, have virtually identical outcomes (Yang et al., 2022). This inefficacy may perhaps be in part due to the lack of personalized seizure network identification, wherein the thalamic nucleus that best disrupts the network may vary between patients. Similarly, a network approach may serve to better understand and better utilize vagal nerve stimulation (VNS). Currently relegated to an adjuvant therapy in generalized DRE, VNS has been FDA approved for over 25 years yet still the mechanism of action remains unclear (Carron et al., 2023). It is believed that VNS modulates a complex network of communication between the brainstem and cerebrum, desynchronizing epileptiform activity (Hachem et al., 2018). Studies have demonstrated that modulation of specific network connections, such as thalamocortical connections, are associated with higher response rates to VNS, underscoring the importance of targeting the entire seizure network for an effective therapeutic response. These studies underline the need to assess the epileptic network and tailor the stimulation site of neuromodulation to effectively perturb the personalized network at large.

**FIGURE 1:**
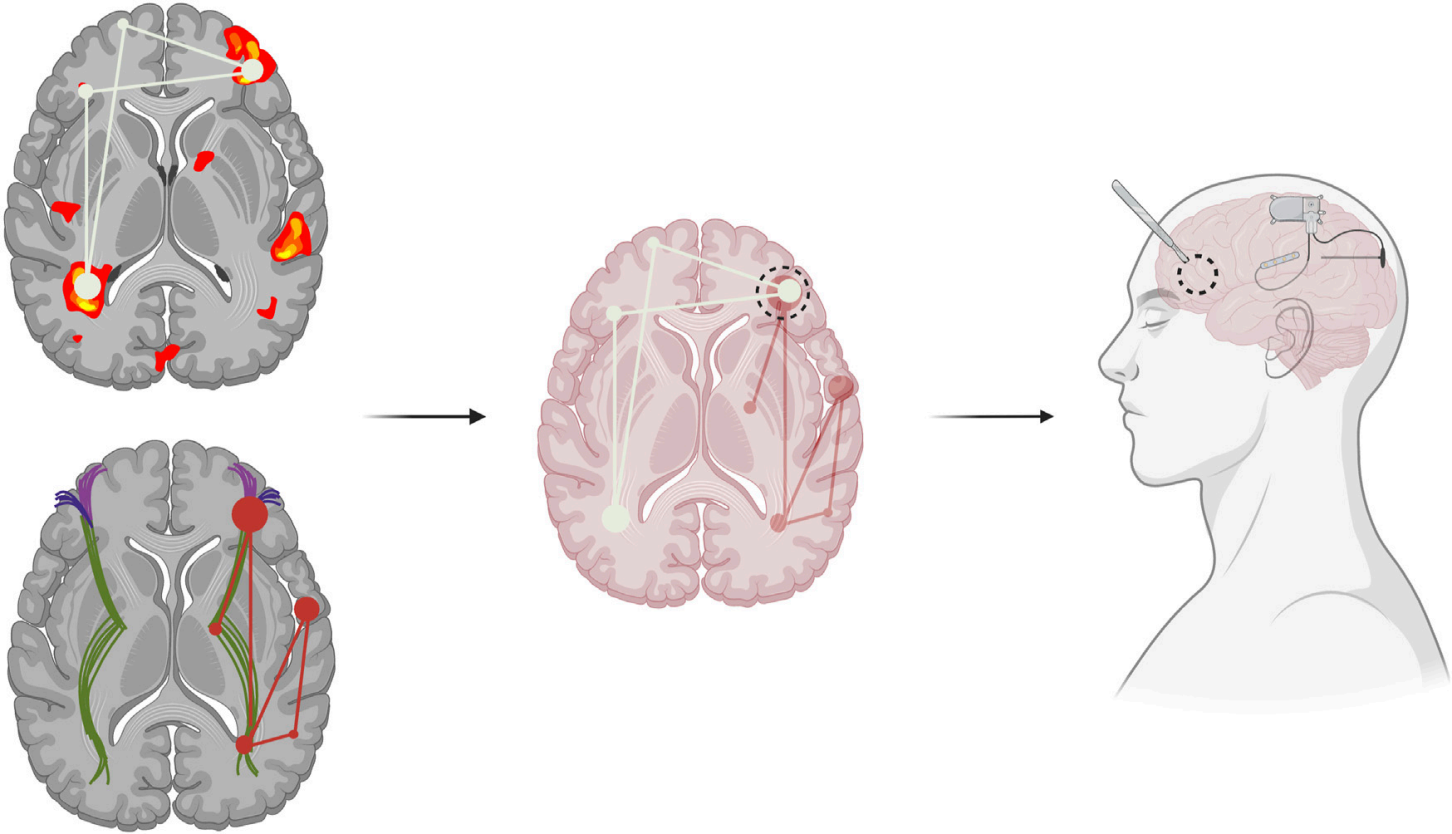
Functional (top left) and structural (bottom left) connectivity can identify seizure networks. Combining various imaging modalities can help locate relevant seizure network hubs, central areas of seizure network activity or communication, which can inform pre-surgical epilepsy planning (middle). Modification of current surgical techniques to more precisely target the identified epileptic network, such as through combined resection with neuromodulation, including the use of the Responsive Neurostimulation System (RNS), of additional seizure network foci (right).

Modifying current pre-surgical evaluations may be the next step in a network-guided approach. For instance, current sEEG mapping involves detecting a seizure focus, but instead, efforts should be shifted more broadly to emphasize identification of the network (Piper et al., 2022). One recent paper reported using sEEG data to construct a seizure network model to identify network-relevant surgical targets for RNS, LITT, and resection in patients with focal epilepsy (Peng et al., 2023). They defined seizure network nodes as the highest ranked sEEG contact points by linear discriminant analysis, a supervised machine learning algorithm. They then described the seizure network as the connections between these nodes, as calculated by DTF-based edge assignment. This model was tested on 10 patients retrospectively, demonstrating both consistency between the model’s recommendations and the clinical decisions that were made. This supports the use of network models to aid in identification of relevant network nodes to augment therapeutic efficacy. Recent studies have supported the use of novel modality combinations, such as hybrid PET/MRI/MEG, to improve the detection of epileptic foci in patients with focal DRE (Guo et al., 2024). Additionally, using advanced techniques, such as graph theory, might unveil particular nodes of significance within the network (Piper et al., 2022). Graph theory utilizes mathematical techniques to characterize structural and functional connections of brain regions at multiple topographical levels (Bernhardt et al., 2015). While few studies have explored the clinical applicability of graph theory analyses based on ECoG recordings to characterize the seizure network, preliminary studies have found evidence of the power of connectivity techniques in predicting post-surgical outcomes for patients with focal epilepsy (Wilke et al., 2011). This suggests the potential utility of structural and functional connectivity analyses which may be able to determine not only areas of connectivity to epileptogenic zones but also the dynamics of how these regions influence one another in the broader context of the network (Figure 1). A multimodal imaging and analytical approach may provide additional insights into the epileptogenic network and may be useful for pre-surgical planning in the future. Once a comprehensive picture of the seizure network is identified, surgical targets may shift from the removal of the seizure onset zone, and with it the entire lobe or implicated region of the brain, and instead towards removal of designated seizure network nodes, or locations that are crucial to sustaining the pathologic network. In this way, network-based epilepsy surgery may minimize damage to healthy brain tissue while simultaneously improving patient outcomes by specifically targeting the critical nodes within the network that propagate seizures.

Advances in surgical techniques may augment network-based surgical targeting. For instance, while resective surgery remains the primary treatment modality for focal epilepsy, patients with multiple seizure foci may warrant a complementary approach with responsive neurostimulation in these critical brain regions inadequately impacted by resection (Salama et al., 2024). One retrospective study has found an improved seizure reduction of 81 % with combinatory resection and responsive neurostimulation, though certainly further studies are warranted to confirm these findings (Tran et al., 2020). This enables intervention of multiple critical regions of the network, even ones distally located from the seizure onset zone. Armed with a network-based understanding of epilepsy, modifying treatment to align with this pathophysiology offers an exciting potential opportunity to improve patient outcomes and advance the field of personalized epilepsy care.

## 5 Conclusion

Epilepsy research over the last few decades has featured a paradigm shift in the pathophysiological understanding of epilepsy from a focal to a network-based disorder. Despite increasing evidence supporting the concept of seizure networks, these findings have not translated to adaptation of current medical and surgical management. Recent studies have implicated a failure to sufficiently modulate the epileptic network as a potential reason for the inefficacy of both pharmacotherapeutic and surgical treatments. Identifying new ways to elucidate the seizure network, such as through combinations of imaging techniques to pinpoint critical nodes, may be useful to help guide presurgical planning. Modification of current surgical techniques with a network-based approach may offer significant strides in seizure freedom rates and improved quality of life for patients with drug-resistant epilepsy.
